# Genetic variation in the vitamin D pathway *CYP2R1* gene predicts sustained HBeAg seroconversion in chronic hepatitis B patients treated with pegylated interferon: A multicenter study

**DOI:** 10.1371/journal.pone.0173263

**Published:** 2017-03-15

**Authors:** Kessarin Thanapirom, Sirinporn Suksawatamnuay, Wattana Sukeepaisarnjareon, Tawesak Tanwandee, Phunchai Charatcharoenwitthaya, Satawat Thongsawat, Apinya Leerapun, Teerha Piratvisuth, Rattana Boonsirichan, Chalermrat Bunchorntavakul, Chaowalit Pattanasirigool, Bubpha Pornthisarn, Supot Tantipanichtheerakul, Ekawee Sripariwuth, Woramon Jeamsripong, Teeranan Sanpajit, Yong Poovorawan, Piyawat Komolmit

**Affiliations:** 1 Division of Gastroenterology, Department of Medicine, Faculty of Medicine, Chulalongkorn University and King Chulalongkorn Memorial Hospital, Thai Red Cross Society, Bangkok, Thailand; 2 Department of Medicine, Faculty of Medicine, Khon Kaen University, Khon Kaen, Thailand; 3 Division of Gastroenterology, Department of Medicine, Siriraj Hospital, Bangkok, Thailand; 4 Department of Internal Medicine, Chiang Mai University, Chiang Mai, Thailand; 5 Faculty of Medicine, Prince of Songkla University, Songkhla, Thailand; 6 Faculty of Medicine, Vajira Hospital, Bangkok, Thailand; 7 Faculty of Medicine, Rajavithi Hospital, Bangkok, Thailand; 8 Faculty of Medicine, Police General Hospital, Bangkok, Thailand; 9 Faculty of Medicine, Thammasat University Hospital, Bangkok, Thailand; 10 Faculty of Medicine, Bhumibol Adulyadej Hospital, Bangkok, Thailand; 11 Faculty of Medicine, Naresuan University, Phitsanulok, Thailand; 12 Faculty of Medicine, Buddhachinaraj Hospital, Phitsanulok, Thailand; 13 Phramongkutklao Hospital, Bangkok, Thailand; 14 Center of Excellence in Clinical Virology, Department of Pediatrics, Faculty of Medicine, Chulalongkorn University, Bangkok, Thailand; Kaohsiung Medical University, TAIWAN

## Abstract

Evidence of a role of vitamin D in the immune system is increasing. Low serum vitamin D is associated with increased hepatitis B virus replication. Genome-wide association study (GWAS) data has revealed a number of the single nucleotide polymorphisms (SNPs) within the vitamin D synthetic pathway that affect vitamin D functions. We aimed to determine the association between SNPs in the vitamin D gene cascade and response to pegylated interferon (PegIFN) therapy in hepatitis B e-antigen (HBeAg)-positive patients. One hundred and eleven patients treated for 48 weeks with PegIFN-alfa 2a at 13 hospitals were retrospectively evaluated. Thirteen SNPs derived from vitamin D cascade-related genes, including *DHCR7* (rs12785878), *CYP27B1* (rs10877012), *CYP2R1* (rs2060793, rs12794714), *GC* (rs4588, rs7041, rs222020, rs2282679), and *VDR* (*Fok*I, *Bsm*I, *Tru*9I, *Apa*I, *Taq*I), were genotyped. Thirty-one patients (27.9%) seroconverted to HBeAg after 24 weeks of treatment. Multivariate analysis found pretreatment qHBsAg <10,000 IU/mL (OR = 7.73, 95% CI: 2.36–25.31, *P* = 0.001), *CYP2R1* rs12794714 TT genotype (OR = 4.16, 95% CI: 1.07–16.25, *P* = 0.04), and baseline ALT >2 times the upper limit of normal (OR = 3.83, 95% CI: 1.31–11.22, *P* = 0.014) predicted sustained HBeAg seroconversion after completion of PegIFN treatment. HBV DNA during study period tended to be lower with the rs12794714 *CYP2R1* TT than the non-TT genotype. The rs12794714 *CYP2R1* polymorphism may be a useful pretreatment factor predictive of sustained HBeAg seroconversion after PegIFN therapy. This study provides evidence that not only vitamin D level but also genetic variation of *CYP2R1* in the vitamin D cascade influences host immune response in chronic HBV infection.

## Introduction

Chronic hepatitis B (CHB) infection is a major global health problem, and more than 350 million persons are chronic carriers of hepatitis B virus (HBV) [1, 2]. Currently available drugs include nucleos(t)ide analogs with high antiviral activity and HBV DNA suppression [3], but most patients will require life-long therapy [4]. Finite duration of treatment with pegylated interferon (PegIFN) achieves an immune response to control HBV infection and induces a sustained response in 20–32% of CHB patients [5–7]. However, this therapy has frequent side effects in addition to a limited response. Therefore, identification of potential baseline predictors of durable off-treatment response is essential. In Hepatitis B early antigen (HBeAg)-positive patients, HBV genotype A or B, high baseline alanine aminotransferase (ALT) level, low pretreatment HBV-DNA level (<2.0×10^8^ IU/mL), female sex, older age, basal core promoter and precore mutations of HBV, and absence of prior PegIFN therapy have been shown to predict a sustained response to PegIFN therapy [8, 9].

In addition to regulating calcium metabolism and bone homeostasis, vitamin D also has an important immunomodulatory effect on innate and adaptive immune responses [10, 11]. Most vitamin D is synthesized from 7-dehydroxycholesterol in sun-exposed skin; some is provided by the diet. The *DHCR7* gene encodes a reductase that catalyzes the conversion of 7-dehydrocholesterol to cholesterol. Dietary or cutaneous vitamin D undergoes two modification steps to become 1, 25-dihydroxyvitamin D [1,25(OH)_2_D], the active form of vitamin D. The first step is performed in the liver by CYP2R1 and generates 25-hydroxyvitamin D [25(OH)D], which is the main circulatory form and is transported while bound to vitamin D binding protein (GC-globulin) [12, 13]. In the second step, 25(OH)D is converted to 1,25(OH)_2_D in the kidney by 1α-hydroxylase. The *CYP27B1* gene encodes 1α-hydroxylase. 1,25(OH)_2_D exerts its biological functions by binding to and activating the vitamin D receptor (VDR) [11].

Low serum 25(OH)D is associated with various cancers, autoimmune, and infectious diseases [14–16], and vitamin D deficiency has been reported in chronic liver disease such as chronic viral hepatitis and nonalcoholic steatohepatitis [17, 18]. In patients with CHB infection, low serum 25(OH)D has been associated with high levels of HBV replication and found to have an inverse relationship with serum HBV DNA level [17]. In addition, HBeAg-positive patients were shown to have lower serum 25(OH)D levels than HBeAg-negative patients [17]. Vitamin D deficiency has been related to adverse clinical outcomes in CHB-infected patients [19] and low baseline vitamin D to ALT normalization after 48 weeks of therapy [20]. A recent genome-wide association study (GWAS) and a systematic review found that variants of genes controlling vitamin D synthesis and transport influenced serum 25(OH)D levels. These included 7-dehydrocholesterol reductase-*DHCR7*, 1-α-hydroxylase*-CYP27B1*, Cytochrome P450, family 2, subfamily R, polypeptide 1-*CYP2R1*, vitamin D binding protein-*GC* and vitamin D receptor-*VDR* [21–24].

There are no data on the association of functional genetic variation of the vitamin D-related genes and outcome of PegIFN therapy in patients with HBeAg-positive CHB. This study aimed to determine the association between the single nucleotide polymorphisms (SNPs) of vitamin D cascade and response to PegIFN therapy in patients with HBeAg-positive CHB infection.

## Materials and methods

### Study participants

We retrospectively analyzed 111 patients with HBeAg-positive CHB infection who were included in the hepatitis B database of the Thai Association for the Study of the Liver (THASL) between January 2010 and December 2011. The patient sample was selected from 13 tertiary hospitals in Thailand, including King Chulalongkorn Memorial Hospital, Chulalongkorn University (Bangkok), Siriraj hospital, Mahidol University (Bangkok), Srinagarind hospital, Khon Kaen University (Khon Kaen), Maharaj Nakorn Chiang Mai hospital, Chiang Mai University (Chiang Mai), Songklanagarind hospital, Prince of Songkla University (Songkhla), Police hospital (Bangkok), Vajira Hospital, Navamindradhiraj University (Bangkok), Rajavithi hospital (Bangkok), Thammasat University hospital (Pathum Thani), Buddhachinaraj Hospital (Phitsanulok), Naresuan University hospital (Phitsanulok), Phramongkutklao hospital (Bangkok) and Bhumibol Adulyadej Hospital (Bangkok).

Eligible patients had been seropositive for HBsAg for more than 6 months before enrollment, seropositive for HBeAg, had a persistent serum ALT level above the upper limit of normal (ULN), and an HBV DNA level > 2,000 IU/mL. Patients previously treated with PegIFN or nucleos(t)ide analogs, with concomitant human immunodeficiency, hepatitis C or hepatitis D virus infection, decompensated cirrhosis, or contraindicated for PegIFN administration were excluded. Patients meeting the entry criteria were treated with subcutaneous PegIFN alfa-2a 180 μg/week (Pegasys, Roche Holding AG, Switzerland) for 48 weeks. Patient data was collected using standard case record forms. Demographic data were recorded as shown in Table 1. Serum ALT, HBV DNA, quantitative HBsAg (qHBsAg), HBeAg and anti-HBe were recorded at baseline, during treatment, and 24 weeks after completion of treatment. The study protocol, consent procedure was approved by the Institutional Review Boards of each study sites and conducted in compliance with principles of the Declaration of Helsinki under Good Clinical Practice. Individuals were informed of the study purpose and written consents were obtained following the requirements of the local ethics committees. Informed consent was signed by the subject or the subject's legally authorized representative.

**Table 1:** Baseline characteristics of patients with HBeAg-positive chronic hepatitis B infection after 48 weeks of PegIFN therapy.

	No sustained HBeAg seroconversion (n = 80)	Sustained HBeAg seroconversion(n = 31)	p-value
**Male, n (%)**	59 (72.8%)	20 (66.7%)	0.64
**Age (years), mean ± SD**	39.1 ± 11.3	42.5 ± 11.2	0.13
**Body mass index (kg/m**^**2**^**), mean ± SD**	23.6 ± 3.6	24.0 ± 3.2	
**HBV genotype, n (%)**			
**B**	12 (15.8%)	1 (3.4%)	
**C**	63 (82.9%)	28 (96.6%)	0.09
**D**	1 (1.3%)	0	
**Cirrhosis, n (%)**	6 (7.7%)	3 (11.1%)	0.13
**Pre-treatment HBV DNA (logIU/ml), mean ± SD**	7.3 ± 1.3	6.6 ± 1.5	0.006
**Pre-treatment HBsAg level (logIU/ml), mean ± SD**	3.9 ± 1.0	3.5 ± 0.7	0.004
**Pre-treatment ALT (U/L), mean ± SD**	95.7 ± 76.1	122.3 ± 91.8	0.04

PegIFN, pegylated interferon; HBV, hepatitis B virus; ALT, alanine aminotransferase; SD, standard deviation.

Sustained HBeAg seroconversion was defined as negative for HBeAg and seropositive for anti-HBe 24 weeks after the end of therapy. Liver cirrhosis was determined by tissue histology, imaging, or measurement of liver stiffness. Loss of HBsAg was defined as undetectable HBsAg (i.e., HBsAg <0.05 IU/mL).

### Laboratory measurement and characterization of SNPs

Thirteen functional SNPs in five genes, which have been reported the association with serum vitamin D from GWAS studies and systematic reviews were genotyped, including *DHCR7* (rs12785878 G>T), *CYP27B1* (rs10877012 C>A), *CYP2R1* (rs2060793 T>C, rs12794714 C>T), *GC* (rs4588 C>A, rs7041 G>T, rs222020 G>A, rs2282679 A>C) and *VDR (Fok*I rs2228570 T>C, *Bsm*I rs1544410 G>A, *Tru*9I rs757343 G>A, *Apa*I rs7975232 G>T, *Taq*I rs731236 T>C)[21–24].

Human genomic DNA was extracted from 100 μl samples of peripheral blood mononuclear cells incubated with proteinase K in lysis buffer, followed by phenol–chloroform extraction, and ethanol precipitation. The pellet was dissolved in 50 μl sterile water and stored at −20°C until further testing. For amplification, a PCR-specific primer set was designed. Aliquots containing 4 μl DNA were diluted 25 μl for PCR assays using Perfect *Taq* plus MasterMix (5 PRIME GmbH, Hamburg, Germany). The primer sequences and PCR conditions are summarized in S1 Table. Amplified DNA (10 μL) in a total volume of 20 μl was digested overnight with two units of restriction endonuclease (New England Biolabs, Hitchin, UK) using the buffers and temperatures recommended by the manufacturer. The resulting DNA fragments were separated electrophoretically on 2% agarose gels. The restriction fragment length polymorphism results were visualized under ultraviolet light after staining with ethidium bromide.

qHBsAg, HBeAg, anti-HBs, anti-HBe were assayed by electrochemiluminescence using an Elecsys (Roche Diagnostics, Indianapolis, IN, USA) or Architect (Abbott Diagnostics, Abbott Park, IL, USA) autoanalyzer. Serum HBV DNA level was quantified using the Roche Cobas Taqman assay, Roche Cobas Amplicor assay, or Abbott RealTime assay. HBV genotype was determined by the INNO-LiPA line probe assay (Innogenetics, Ghent, Belgium).

### Statistical analysis

Statistical analysis was performed using SPSS software (version 22.0; IBM, New York city, NY, USA). The associations between variables were assessed using the Fisher’s exact test for categorical variables, and the Mann–Whitney test for continuous variables. Predictors of sustained HBeAg seroconversion were identified using multivariate logistic regression analysis. A *P*-value <0.05 was considered statistically significant for multiple logistic regression analysis. While Bonferroni Correction was used to adjust p-value for factors, which were compared multiple times including qHBsAg and HBV DNA. The assumption of Hardy-Weinberg equilibrium was assessed for all SNPs using χ^2^ test.

## Results

### Patient characteristics and prevalence of the studied SNPs

A total of 111 patients with HBeAg-positive CHB infection were enrolled; 71.2% (n = 79) were men, and the mean age was 39.9 ± 11.3 years. All patients were Thai; nine (8.6%) had compensated cirrhosis, and 91 (86.7%) were infected with HBV genotype C. Median pretreatment HBV DNA was 7.64 log IU/mL, qHBsAg was 3.93 log IU/ml, and ALT 75 was IU/ml. Table 1 shows the baseline characteristics of patients with and without HBeAg seroconversion at 24 weeks after treatment completion.

The genotypic distribution of the SNPs in this study is shown in Table 2. Genotypic distribution of all alleles were in Hardy–Weinberg equilibrium equation (P > 0.05).

**Table 2:** Genotype frequencies of *DHCR7*, *CYP27B1*, *CYP2R1*, *GC* and *VDR* in HBeAg-positive chronic hepatitis B patients treated with PegIFN for 48 weeks.

	All patients (n = 111)	Non Sustained HBeAg seroconversion (n = 80)	Sustained HBeAg seroconversion (n = 31)	Odds ratio (95% CI)	p-value
***DHCR7* rs12785878 G>T**					
GG	49 (44.1%)	35 (43.2%)	14 (46.7%)	1.15(0.50–2.67)	0.83
GT	51 (46.0%)	40 (49.4%)	11 (36.7%)		
TT	11 (9.9%)	6 (7.4%)	5 (16.6%)		
***CYP27B1* rs10877012 C>A**					
CC	24 (21.6%)	18 (22.2%)	6 (20.0%)	0.87 (0.31–2.47)	1.00
CA	52 (46.9%)	37 (45.7%)	15 (50.0%)		
AA	35 (31.5%)	26 (32.1%)	9 (30.0%)		
***CYP2R1* rs2060793 T>C**					
TT	12 (10.8%)	11 (13.6%)	1 (3.3%)	0.22 (0.03–1.78)	0.17
TC	39 (35.1%)	29 (35.8%)	10 (33.4%)		
CC	60 (54.1%)	41 (50.6%)	19 (63.3%)		
***CYP2R1* rs12794714 C>T**					
CC	44 (39.6%)	36 (44.5%)	8 (26.7%)		
CT	51 (46.0%)	38 (46.9%)	13 (43.3%)		
TT	16 (14.4%)	7 (8.6%)	9 (30.0%)	4.53 (1.51–13.61)	0.01
***GC* rs4588 C>A**					
CC	55 (49.5%)	41 (50.6%)	14 (46.7%)	0.85 (0.37–1.98)	0.83
CA	50 (45.1%)	34 (42.0%)	16 (53.3%)		
AA	6 (5.4%)	6 (7.4%)	0		
***GC* rs7041 G>T**					
GG	6 (5.4%)	5 (6.2%)	1 (3.3%)	0.52 (0.06–4.68)	1.00
GT	56 (50.5%)	39 (48.1%)	17 (56.7%)		
TT	49 (44.1%)	37 (45.7%)	12 (40.0%)		
***GC* rs222020 G>A**					
GG	29 (26.1%)	21 (25.9%)	8 (26.7%)	1.04 (0.40–2.68)	1.00
GA	47 (42.4%)	35 (43.2%)	12 (40.0%)		
AA	35 (31.5%)	25 (30.9%)	10 (33.3%)		
***GC* rs2282679 A>C**					
AA	59 (53.2%)	43 (53.1%)	16 (53.3%)	1.01 (0.44–2.34)	1.00
AC	46 (41.4%)	32 (39.5%)	14 (46.7%)		
CC	6 (5.4%)	6 (7.4%)	0		
***VDR Fok*I rs2228570 T>C**					
TT	26 (23.4%)	22 (27.2%)	4 (13.3%)	0.41 (0.13–1.32)	0.21
TC	61 (55.0%)	42 (51.8%)	19 (63.3%)		
CC	24 (21.6%)	17 (21.0%)	7 (23.4%)		
***VDR Bsm*I rs1544410 G>A**					
GG	98 (88.3%)	72 (88.9%)	26 (86.7%)	0.81 (0.23–2.87)	0.75
GA	12 (10.8%)	9 (11.1%)	3 (10.0%)		
AA	1 (0.9%)	0	1 (3.3%)		
***VDR Tru*9I rs757343 G>A**					
GG	59 (53.2%)	47 (58.0%)	12 (40.0%)	0.48 (0.21–1.13)	0.09
GA	49 (44.1%)	32 (39.5%)	17 (56.7%)		
AA	3 (2.7%)	2 (2.5%)	1 (3.3%)		
***VDR Apa*I rs7975232 G>T**					
GG	53 (47.8%)	43 (53.1%)	10 (33.3%)	0.44 (0.18–1.06)	0.08
GT	48 (43.2%)	32 (39.5%)	16 (53.4%)		
TT	10 (9.0%)	6 (7.4%)	4 (13.3%)		
***VDR Taq*I rs731236 T>C**					
TT	102 (91.9%)	75 (92.6)	27 (90.0%)	0.72 (0.17–3.08)	0.70
TC	9 (8.1%)	6 (7.4%)	3 (10.0%)		
CC	0	0	0		

PegIFN, pegylated interferon.

### Treatment response and HBV genotype

When the treatment ended, 53 patients (54.1%) had HBV DNA <2,000 IU/ml and ALT normalization occurred in 67 (60.4%). At 24 weeks after PegIFN discontinuation, 30 (27.0%) achieved sustained HBeAg seroconversion, eight (7.5%) cleared HBsAg, and 67 (60.4%) had ALT normalization. The result of HBV genotype was available in 105 patients (94.6%). Table 3 shows the patient responses at the end of treatment and at 24 weeks of follow-up classified according to HBV genotype. Sustained HBeAg seroconversion was obtained in 30.8% (n = 28) of patients with the C genotype and 7.1% (n = 1) of those with the non-C genotype. At 24 weeks after PegIFN discontinuation, 6.8% of patients with the C genotype and 7.1% of those with the non-C genotype were HBsAg negative, and ALT normalization was achieved by 59.3%, 85.7%, respectively. Treatment outcomes were not different between patients with HBV genotype C and non-C at end-of-treatment and 24 weeks of follow-up, except genotype C had more HBeAg seroconversion at end-of-treatment.

**Table 3:** Outcomes in patients with HBeAg-positive CHB infection stratified by HBV genotype after completing 24 weeks of PegIFN treatment.

	Total (n = 111)	HBV genotype C (n = 91)	HBV non-genotype C (n = 14)	p-value
**At end-of-treatment**				
HBV DNA < 2,000 IU/ml	53 (54.1%)	42 (53.2%)	7 (50.0%)	0.83
HBeAg seroconversion	26 (25.2%)	26 (31.0%)	0	0.01
HBsAg loss	5 (5.1%)	5 (6.1%)	0	0.36
ALT normalization	67 (60.4%)	54 (59.3%)	12 (64.3%)	0.06
**At 24 weeks of follow-up**				
HBV DNA < 2,000 IU/ml	42 (41.2%)	36 (43.4%)	5 (35.7%)	0.13
HBeAg seroconversion	30 (27%)	28 (30.8%)	1 (7.1%)	0.07
HBsAg loss	8 (7.5%)	6 (6.8%)	1 (7.1%)	0.96
ALT normalization	67 (60.4%)	54 (59.3%)	12 (85.7%)	0.06

PegIFN, pegylated interferon; HBV, hepatitis B virus; CHB, chronic hepatitis B; ALT, alanine aminotransferase.

### Treatment response in patients with *DHCR7* (rs12785878), *CYP27B1* (rs10877012), *CYP2R1* (rs2060793, rs12794714), *GC* (rs4588, rs7041, rs222020, rs2282679), and *VDR* (*Fok*I, *Bsm*I, *Tru*9I, *Apa*I, *Taq*I) gene polymorphisms

The overall genotype frequencies of the SNPs in vitamin D pathway genes observed in all patients and in HBeAg-positive patients with and without sustained HBeAg seroconversion are summarized in Table 2. The *CYP2R1* rs12794714 TT genotype was associated with a higher rate of sustained HBeAg seroconversion than non-TT genotype (56.2% vs. 22.1%, *P* = 0.01). Patients who achieved HBeAg seroconversion had a higher T-allele frequency of *CYP2R1* rs12794714 than patients who did not achieve that endpoint (51.6% vs. 32.1%, p = 0.007). Loss of HBeAg was observed in nine patients with the *CYP2R1* rs12794714 TT genotype (56.2%) vs. 28 patients with the non-TT genotype (30.1%). The remaining SNPs of the *DHCR7* (rs12785878), *CYP27B1* (rs10877012), *CYP2R1* (rs2060793), *GC* (rs4588, rs7041, rs222020, rs2282679), *VDR* (*Fok*I, *Bsm*I, *Tru*9I, *Apa*I, *Taq*I) genes were not related to HBeAg seroconversion after therapy. The genetic variations of the SNPs in the vitamin D pathway genes were not associated with seroclearance of HBsAg, or ALT normalization at 24 weeks after PegIFN treatment.

### Prevalence of *CYP2R1* rs12794714 and association with sustained HBeAg seroconversion among HBV genotypes

The genotype frequencies of *CYP2R1 rs12794714* CC, CT, and TT were 39.6%, 44.0%, and 16.5%, respectively, in HBV genotype C and 42.9%, 57.1%, and 0%, in HBV non-genotype C patients. The *CYP2R1* rs12794714 TT genotype was strongly associated with HBeAg seroconversion in HBV genotype C patients, where 60.0% with the TT genotype achieved sustained HBeAg seroconversion compared with 25.0% of those with the non-TT genotype (p = 0.007). The *CYP2R1* rs12794714 polymorphism was not associated with HBeAg seroconversion after HBV treatment in non-genotype C patients.

### Pretreatment predictors of sustained HBeAg seroconversion

Following Univariate analysis, potentially confounding variables with *P* ≤ 0.1, including baseline HBV DNA, qHBsAg, ALT, HBV genotype, *CYP2R1* rs12794714 TT, *VDR Apa*I GG, and *VDR Tru9*I GG genotypes were evaluated by multivariate analysis. Pretreatment qHBsAg <10,000 IU/mL (OR = 7.73, 95% CI: 2.36–25.31, *P* = 0.001), *CYP2R1* rs12794714 TT genotype (OR = 4.16, 95% CI: 1.07–16.25, *P* = 0.04), and baseline ALT >2 times the upper limit of normal (OR = 3.83, 95% CI: 1.31–11.22, *P* = 0.014) were independent predictors of sustained HBeAg seroconversion after PegIFN therapy in HBeAg-positive patients with CHB infection.

### Association of HBV DNA and qHBsAg kinetics during and after treatment with *CYP2R1* rs12794714 genotype

**Fig 1:**
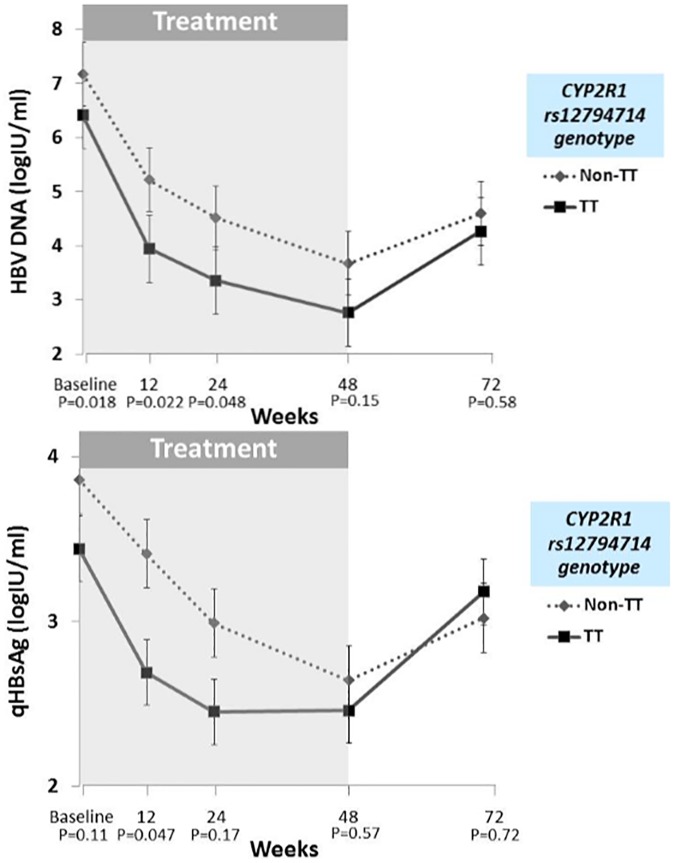
Mean HBV DNA and qHBsAg during PegIFN therapy in patients stratified by *CYP2R1* rs12794714 TT and non-TT genotype.

The kinetics of HBV DNA and qHBsAg during and after PegIFN therapy in patients stratified by *CYP2R1* rs12794714 TT and non-TT genotype are shown in Fig 1. Mean HBV DNA (log IU/ml) tended to be lower in patients with *CYP2R1* rs12794714 TT than in those with non-TT genotype at baseline and during PegIFN therapy. Also, after discontinuation of PegIFN treatment, HBV DNA there was a trend toward continuing suppression TT patients compared with non-TT genotype patients. Mean qHBsAg during and after therapy tended to be lower in TT compared with non-TT *CYP2R1* rs12794714 genotype, but the differences did not reach significance.

## Discussion

In this study, we investigated the possible association between functional genetic polymorphisms in the vitamin D metabolic pathway—*DHCR7*, *CYP27B1*, *CYP2R1*, *GC*, and *VDR*—and sustained treatment response in patients with HBeAg-positive CHB infection treated with PegIFN for 48 weeks. The main finding is that polymorphism of rs12794714 of the *CYP2R1* gene independently predicted sustained HBeAg seroconversion in HBeAg-positive patients after completing PegIFN treatment. This suggests that the PegIFN-influenced immune response may be partially determined by host genetic factors in the vitamin D cascade. PegIFN on its own led to a sustained off-treatment response in approximately one-third of the patients, and was therefore a potent treatment option for patients with HBeAg-positve CHB. Several host and viral factors have been proposed to predict response to PegIFN therapy [8, 25].

Recently, the evidence that vitamin D plays important role in immunomodulation has become stronger [10, 11], and host immune status has been shown to be key for both persistent CHB infection and PegIFN treatment responses [26, 27]. A high prevalence of vitamin D deficiency and insufficiency, ranging from 81–93%, has been seen in patients with CHB infection [17, 20], and serum 25(OH)D concentration has been seen to be significantly lower than in healthy individuals living in the same geographic region [15, 28]. Low serum vitamin D levels have been associated with high levels of HBV replication and poor clinical outcomes, including cirrhotic complications, hepatocellular carcinoma, and death [17, 19]. Previous studies showed that genetic variation of the vitamin D metabolic pathway, including *CYP27B1-1260* rs10877012 and *VDR Fok*I rs2228570, was associated with sustained virological response to PegIFN therapy in patients with chronic hepatitis C infection [29, 30]. However, in patients with CHB infection, polymorphism of the *CYP27B1* SNP rs4646536 promoter was related to PegIFN response in HBeAg-negative patients [31]. In this patient series, the *CYP2R1* rs12794714 TT genotype predicted sustained HBeAg seroconversion after PegIFN treatment. This novel host genetic factor might be useful for predicting treatment response to PegIFN in HBeAg-positive patients. The mechanism was not clearly identified, but we found that patients with the *CYP2R1* rs12794714 TT genotype tended to have lower HBV DNA from baseline to 24 weeks posttreatment follow-up compared with those with a non-TT genotype. In addition, the *CYP2R1* rs12794714 TT genotype had trend to be associated with lower HBV DNA and qHBsAg during and after PegIFN therapy. This genotype variant might be reflective of an active host immune response.

The *CYP2R1* gene, located on chromosome 11p15.2, contains five exons, includes about 15.5 kb, and encodes vitamin D 25-hydroxylase. The rs12794714 SNP of *CYP2R1* was significantly associated with serum 25(OH)D in Han children in northeast China and in Singapore [32, 33]. There have been no prior data of *CYP2R1* rs12794714 genotypic frequencies in Thai populations, but the frequencies were 39.6% for the CC, 48.7% for the CT, and 11.7% for the TT genotypes in middle-aged and elderly Singapore Chinese [33]. This study indicated that not only serum vitamin D level but also the *CYP2R1* rs12794714 variant was related to HBV replication, but that it also predicted response to PegIFN treatment in patients with HBeAg-positive CHB. None of the other 12 SNPs of the vitamin D genes that were evaluated predicted a sustained response to PegIFN therapy.

In addition to *CYP2R1* rs12794714 polymorphism, this study demonstrated that baseline qHBsAg < 10,000 IU/mL and ALT levels > 2 times the upper limit of normal were also pretreatment factors for predicting HBeAg seroconversion in Thai HBeAg-positive patients treated with PegIFN. These findings are similar to those reported by Fan *et al*. showing that baseline anti-HBc, HBV DNA, and ALT were predictors of HBeAg seroconversion in HBeAg-positive patients treated with PegIFN or nucleos(t)ide analogues [34]. In term of selection these cut-off for baseline qHBsAg and ALT level to perform multivariate analysis were based on previous studies [35, 36]. Our study had several limitations. First, we did not evaluate the effect of baseline serum 25(OH)D level because it is influenced by several potentially confounding variables including exposure to UV sunlight, malabsorption, reduced dietary intake of the vitamin, and decreased hepatic hydroxylation resulting from chronic liver disease [25]. Also, we wanted to demonstrate the association of key functional polymorphisms of vitamin D genes with treatment response, which might not be affected by other factors. Second, this study included the small number of HBeAg-positive patients and had limited period of follow-up, which might reduce the precise conclusion and hardly prove that there were no association between other studied SNPs and HBeAg seroconversion. Further studies with large number of patients are needed to clarify the influence of vitamin D related gene polymorphisms on the PegIFN treatment.

## Conclusions

The *CYP2R1* rs12794714 TT genotype may be associated with sustained HBeAg seroconversion and may be a promising baseline predictor of PegIFN response, which help to identify HBeAg-positive CHB patients suitable for PegIFN therapy.

## Supporting information

S1 Table The primer sequences and polymerase chain reaction conditions of 13 studied single nucleotide polymorphisms. (DOC)
