# Supplementary material for: Genetic variation in the vitamin D pathway CYP2R1 gene predicts sustained HBeAg seroconversion in chronic hepatitis B patients treated with pegylated interferon: A multicenter study
Source: PLoS One. 2017 Mar 15;12(3):e0173263. doi: 10.1371/journal.pone.0173263 (PMC5351865; doi:10.1371/journal.pone.0173263)
Supplement: S1 Table — (DOC) [file pone.0173263.s001.doc]

**S1 Table: The primer sequences and polymerase chain reaction conditions of 13 studied single nucleotide polymorphisms.**

| **SNP ID** | **primer sequence** | **Temp. annealing (°C)** | **Restric-tion enzyme** | **allele** | **product size (bp)** |
| --- | --- | --- | --- | --- | --- |
| **rs10877012** | F: 5' TGA CCT TCA ATT CCA GAA CTT CA 3' | 58 | *Hinf*I | C | 151, 104 |
|  | R: 5' GGT GGC GTA TGC CTG TAG TG 3' |  |  | A | 255 |
| **rs2060793** | F: 5' CCT TGA TAT TTC CTC TGT TTG 3' | 58 | *Hinf*I | T | 285 |
|  | R: 5' TCG CTG TCT CTC TGA TTA TCT 3' |  |  | C | 194, 91 |
| **rs12794714** | F: 5' CGC TCT TCC TGC TGC TCT TC  3' | 58 | *Fok*I | C | 240, 110 |
|  | R: 5' GCG TCG AGG ACT TCT CCC TTC 3' |  |  | T | 353 |
| **rs7041** | F: 5' TAC CAC AGG TAT AGA ATT TT 3' | 53 | *Hae*III | G | 183, 121 |
|  | R: 5' AGT GGA GGG TTA CAT TTT CCT 3' |  |  | T | 340 |
| **rs4588** | F: 5' TAC CAC AGG TAT AGA ATT TT 3' | 53 | *Btg*I | C | 190, 114 |
|  | R: 5' AGT GGA GGG TTA CAT TTT CCT 3' |  |  | A | 340 |
| **rs222020** | F: 5' TTT GAC GTT AGG GCT TCA TCA ACT CAA TGG GCA AAA AAT TCA ATC G 3' | 57 | *Taq*I | G | 330 |
|  | R: 5' AGC GAG CTG GCT AGG AGT TGT C 3' |  |  | A | 286, 44 |
| **rs2282679** | F: 5' AGT AAT ACC TAC AAT TCA TGT 3' | 52 | *Fok*I | A | 425, 126 |
|  | R: 5' AGC TAA CAA TAA AAA ATA CCT GGA T 3' |  |  | C | 425, 126, 69, 39 |
| **rs2228570** | F: 5' TGG CAC TGA CTC TGG CTC TGA 3' | 58 | *Fok*I | T | 205, 61 |
|  | R: 5' CTC CCT TCA TGG AAA CAC CTT G 3' |  |  | C | 266 |
| **rs1544410** | F: 5' CTC ACT GCC CTT AGC TCT GC 3' | 58 | *Bsm*I | G | 254, 103 |
|  | R: 5' TTG GAC CTC ATC ACC GAC AT 3' |  |  | A | 357 |
| **rs757343** | F: 5' CTC ACT GCC CTT AGC TCT GC 3' | 58 | *Tru*9I | G | 357 |
|  | R: 5' TTG GAC CTC ATC ACC GAC AT 3' |  |  | A | 264, 94 |
| **rs7975232** | F: 5' TTT GGG GCC AGG CAG TGG TAT 3' | 58 | *Apa*I | G | 229, 111 |
|  | R: 5' CGG TAC TGC TTG GAG TGC TCC TC 3' |  |  | T | 340 |
| **rs731236** | F: 5' TTT GGG GCC AGG CAG TGG TAT 3' | 58 | *Taq*I | T | 340 |
|  | R: 5' CGG TAC TGC TTG GAG TGC TCC TC 3' |  |  | C | 191, 149 |
| **rs12785878** | F: 5' CTG TCT TCT CTT AGG AGG TT 3' | 56 | *Taq*I | G | 250, 124 |
|  | R: 5' CAA GCA GCA GAC AGG ACA TGA 3' |  |  | T | 374 |
